# No inter-gyre pathway for sea-surface temperature anomalies in the North Atlantic

**DOI:** 10.1038/ncomms11333

**Published:** 2016-04-22

**Authors:** Nicholas P. Foukal, M. Susan Lozier

**Affiliations:** 1 Nicholas School of the Environment, Duke University, Durham, North Carolina 27708, USA

## Abstract

Recent Lagrangian analyses of surface drifters have questioned the existence of a surface current connecting the Gulf Stream (GS) to the subpolar gyre (SPG) and have cast doubt on the mechanism underlying an apparent pathway for sea-surface temperature (SST) anomalies between the two regions. Here we use modelled Lagrangian trajectories to determine the fate of surface GS water and satellite SST data to analyse pathways of GS SST anomalies. Our results show that only a small fraction of the surface GS water reaches the SPG, the water that does so mainly travels below the surface mixed layer, and GS SST anomalies do not propagate into the SPG on interannual timescales. Instead, the inter-gyre heat transport as part of the Atlantic Meridional Overturning Circulation must be accomplished via subsurface pathways. We conclude that the SST in the SPG cannot be predicted by tracking SST anomalies along the GS.

## Abstract (web summary)

It has been proposed that sea-surface temperature (SST) anomalies along the Gulf Stream aid regional climate predictions months to years in advance. Here, the authors show that a surface pathway for SST anomalies does not exist, as Gulf Stream waters reach higher latitudes by sub-surface pathways.

## Graphical abstract

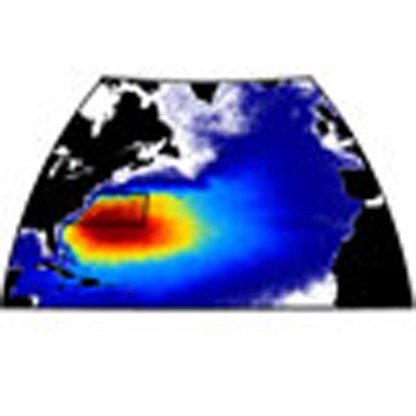

The surface waters of the eastern subpolar gyre (50° N–60° N and 30° W–15° W) in the North Atlantic are exceptionally warm and salty compared with the surface waters at similar latitudes, leading to the supposition that the subpolar gyre (SPG) is primarily supplied by the similarly warm and salty waters of the subtropical gyre (STG)^1^,^2^,^3^. This connection has been of particular interest because the sea-surface characteristics of the North Atlantic SPG provide the environment for density-driven overturning—the warm surface water loses heat to the atmosphere, becomes denser and sinks to depth as it flows around the SPG. This water mass transformation is one component of the Atlantic Meridional Overturning Circulation (AMOC), which moves warm water northwards in its upper layers and colder water southwards at depth, affecting regional and global climate by redistributing heat^4^. Another impetus for understanding the structure and pathways of the upper-layer AMOC is the potential for predicting climate on timescales of months to years in advance^5^,^6^,^7^.

Eulerian-based analyses of surface drifter velocities—in which drifter velocities within a spatially defined bin are averaged over time—indicate that surface waters in the Cape Hatteras region are advected north-eastward into the eastern SPG^3^,^8^. In contrast, Lagrangian analyses of the pathways of individual trajectories from the same surface drifter data show little to no surface connection between the Gulf Stream (GS) and the eastern SPG^9^,^10^. Only 1 out of 273 (0.37%) surface drifters deployed south of 45° N in the North Atlantic between 1990 and 2002 made it to the SPG^9^. Though this surface throughput has increased since 2002, the largest exchange observed during any given time period is only 3.3% (ref. 10). A subsequent modelling study demonstrates that Lagrangian trajectories initialized in the STG at depth shoal as they move northward along isopycnal surfaces and are more likely to reach the SPG than those initialized at the surface in the STG^11^. When coupled with recent findings on the formation and fate of subtropical mode water (STMW)^12^,^13^, these Lagrangian results yield a new circulation pattern of North Atlantic upper-layer currents: surface GS water recirculates in the STG, becomes part of the STMW, re-enters the GS at depth and is then exported to the eastern SPG^14^.

This proposed paradigm, with subsurface STG waters supplying the eastern SPG, begs the question as to whether winter sea-surface temperature (SST) anomalies are transmitted to the SPG via the advective pathway described in previous studies^5^,^15^,^16^ in which SST anomalies re-emerge during subsequent winters as they progress along the GS/North Atlantic Current (GS/NAC). The goal of this paper is to answer that question by first carefully analysing and quantifying the fate of surface GS waters and second, determining whether winter SST anomalies in the modern satellite SST record propagate from the STG into the SPG.

Here we find that only a small fraction of surface GS water reaches the SPG, and that the water that does reach the SPG does so at depths below the winter-mixed layer and is thus out of contact with the SST. We also find that the SST anomaly pathway previously reported is most likely an artefact of a smoothing filter on the same timescale as SST changes associated with the North Atlantic Oscillation (NAO), and not an actual physical pathway for SST anomalies. We conclude that interannual changes in the SST of the SPG cannot, as once thought, be predicted from information on the GS SST and that inter-gyre heat transport of the AMOC must occur below the surface layers.

## Results

### Modelled Lagrangian pathways

**Figure 1:**
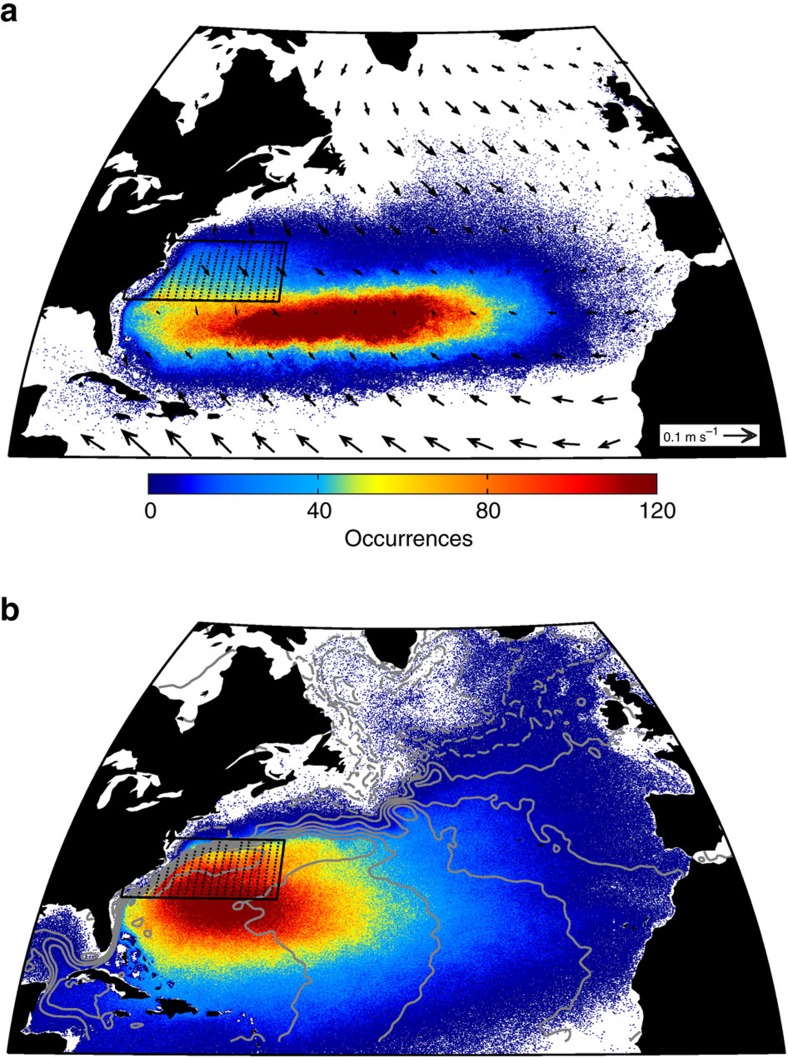
Distributions of modelled particle positions. Floats are initialized from the surface of the VCH box (black rectangle) in FLAME and are constrained to follow either the two-dimensional surface flow field (**a**) or the three-dimensional flow field (**b**) for 5 years. The colour bar refers to the total number of trajectories that pass through a given location (that is, occurrences) over the 15 years. Arrows in **a** are the time-mean Ekman velocities from the Center for Topographic Studies of the Ocean and Hydrosphere^28^ and contours in **b** are the time-mean SSH values from AVISO^29^ (dashed contours indicate negative values).

To trace the pathways and fate of surface GS waters, we initialize Lagrangian particles in an eddy-resolving ocean circulation model (FLAME)^17^,^18^. FLAME is an ocean-only, fully dynamic model with 1/12° horizontal resolution and 45 depth layers varying from 5-m resolution at the surface to 250-m resolution at depth. The model is forced by NCEP-NCAR reanalysis wind and buoyancy forcing for 15 years (1990–2004). We find Lagrangian particle positions using the model's 3-day velocity fields, and record those positions every 15 days. Particles are initialized at the surface in the vicinity of Cape Hatteras (VCH) in a spatial box defined as in a previous study^5^ (31.5° N–38.5° N and 80° W–60° W). We also require that the particles' initial positions be located south of the GS north wall—defined to be the 15 °C isotherm at 200-m depth^19^—so that all trajectories are of subtropical origin. To calculate particle pathways from the model's velocity field, we initially use only horizontal velocities—that is, we restrict the simulated floats to the surface to mimic the constraint placed on the observational surface drifters (Fig. 1a). In a subsequent Lagrangian experiment, we use the three-dimensional flow field (Fig. 1b) for the trajectory calculations. In both experiments, water particles are tagged at the surface in the VCH box every 15 days for the first 10 years of the model run (1990–1999) and tracked for 5 years. In the surface-constrained run, the trajectories are heavily influenced by the winds, accumulating in the Ekman convergence zone at 30° N. Only 5 out of 60,906 modelled trajectories (<0.01%) cross north of 53° N, the latitude at which we consider the floats to be subpolar. When allowed to follow the three-dimensional flow field, the vast majority of the trajectories (97.5%) trace the wind-driven, western-intensified geostrophic STG, accumulating in its convergent centre. Only 1,526 out of the 60,906 total floats (2.51%) reach the SPG in this experiment. Along with vertical mixing, Ekman pumping from negative wind-stress curl forcing yields a net downward transport in the STG^20^, removing particles from the surface layer and creating the disparity between a and b in Fig. 1. These results agree with the observed and modelled Lagrangian experiments on the fate of surface water in the GS^9^,^10^,^11^,^21^, and we elaborate on those previous studies by exploring the pathways of the Lagrangian particles from the GS region to the SPG.

**Figure 2:**
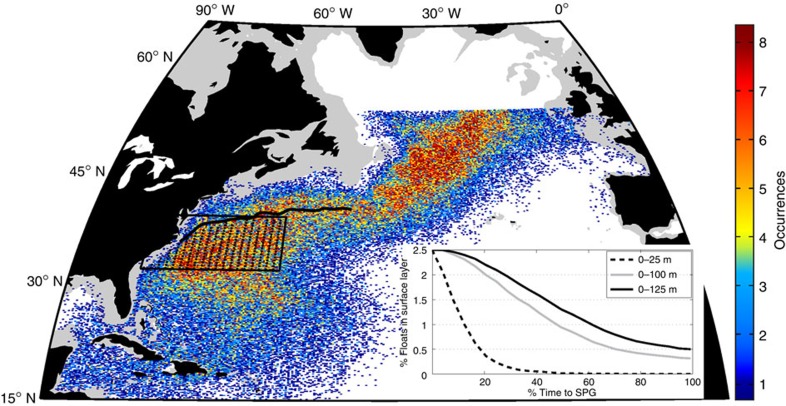
Distribution of modelled particle positions that are SPG-bound. Particles that reach 53° N within 5 years in the three-dimensional flow field (2.51% of total). The colour bar refers to the total number of trajectories that passed through a given location (that is, occurrences) over the 15 years. Particle locations north of 53° N are not displayed. Solid black line indicates the average position of the GS north wall in FLAME. Inset panel shows the percent of trajectories that remain in the surface layer as a function of time, normalized by the total time required to reach the SPG. Three definitions of the surface layer are provided (0–25, 0–100 and 0–125 m).

**Figure 3:**
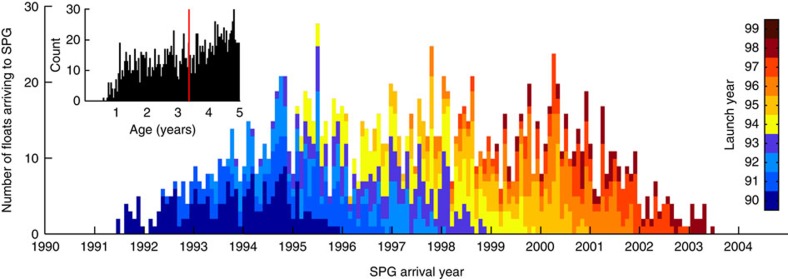
Number of modelled particles crossing 53° N every 30 days. Colours denote the year of initialization in the VCH box. Inset panel shows the distribution of float ages when crossing 53° N, with a median (red line) at 3.37 years.

An examination of those trajectories that reach the SPG within their 5-year lifetime reveals the canonical GS/NAC pathway (Fig. 2). However, only 0.5% of the floats remain above the 125-m climatological winter-mixed layer in the GS/NAC^22^ on their way to the SPG (Fig. 2, inset). In addition, the small number of particles that travel to the eastern SPG vary in their transit time (median=3.4 years, s.d.=1.2 year); the spread demonstrates that waters from the VCH do not travel coherently to the eastern SPG and instead mix with waters of other ages (Fig. 3). In comparison, the Lagrangian integral timescale of temperature anomalies for the trajectories that reach the SPG (∼48 days) is much shorter than the advective timescale, thus individual trajectories are not expected to carry their temperature anomaly to the SPG.

### Satellite SST anomaly pathways

**Figure 4:**
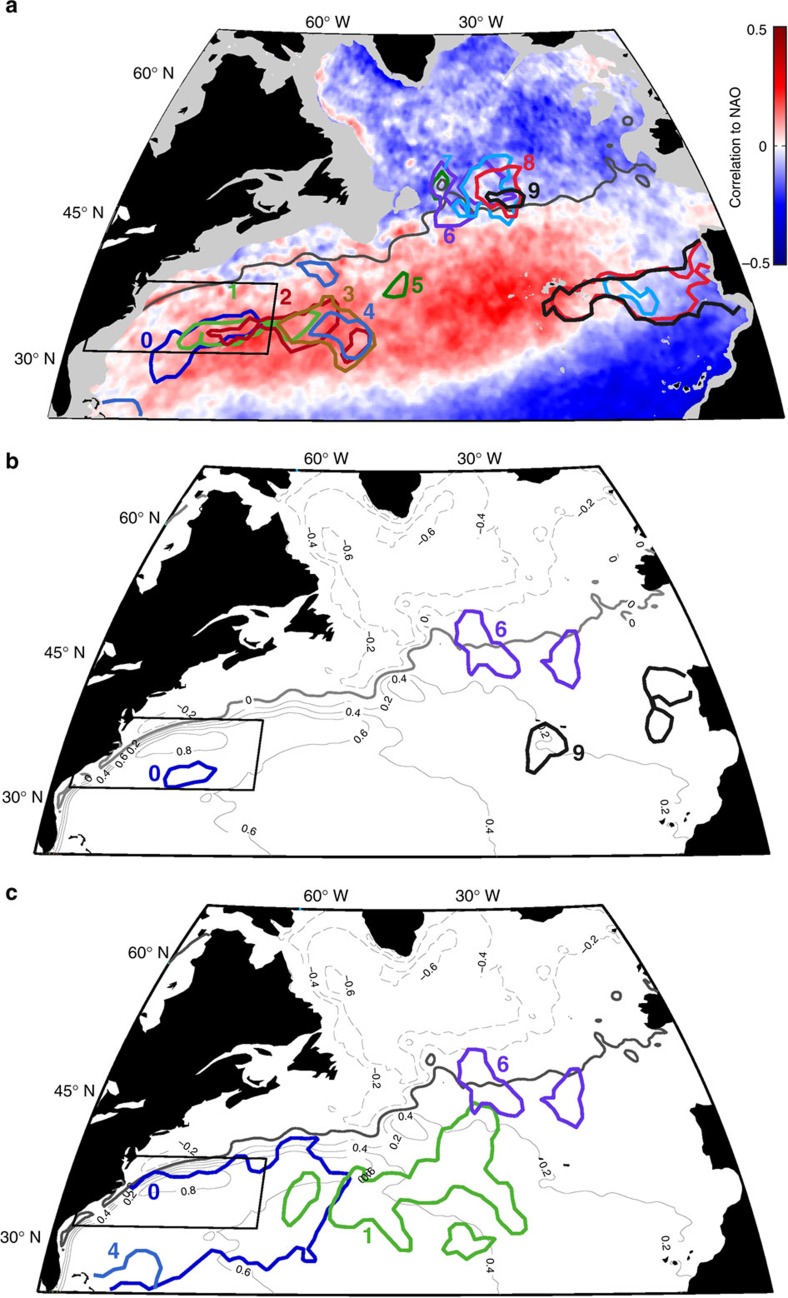
Lagged correlation analysis with the modern satellite SST record. Correlations are calculated between the local SST and the time series from the spatially averaged VCH box (black rectangle). (**a**) Correlations that exceed subjectively chosen thresholds that decrease as the lag increases (lag 0=0.8, lag 1=0.75, lag 2=0.65, lag 3=0.55, lag 4=0.50, lag 5=0.40, lag 6=0.40, lag 7=0.40, lag 8=0.35 and lag 9=0.35). The thresholds used in lags 7 through 9 are not statistically significant (*P*>0.05). Winter SST anomalies are smoothed with a 5-year running mean before calculating correlations. Correlations between the NAO and North Atlantic SST from 1981–2012 are shown in the background. Bathymetry above 1,000 m is shaded light grey, and the dark grey line represents the zero SSH contour. (**b**) Correlation contours are calculated as in **a** but the 5-year running mean filter is removed. (**c**) Objectively chosen, statistically significant (*P*<0.05) correlation contour thresholds with no running mean filter. In **b** and **c**, time-mean AVISO SSH (1993–2014) contours (metres) are shown in grey. An identical analysis with March SST yields qualitatively similar results.

Given the lack of a surface advective pathway from the STG to the SPG, we revisit the question of whether winter SST anomalies propagate along the GS/NAC^5^,^15^,^16^. Our investigation uses a lagged correlation analysis with the modern satellite SST record—31 years (1981–2012) of AVHRR Pathfinder SST data^23^. We switch to observational data for this analysis because a reliable data set exists with an observational period long enough for this analysis. A number of steps are followed to provide consistency with previous studies: (1) we use winter (November–April mean) SST anomalies at 1° × 1° spatial resolution; (2) the VCH box is defined as 80° W–60° W and 31.5° N–38.5° N; and (3) we apply a 5-year running mean filter to the SST anomalies before calculating correlations. A time series of winter SST anomalies averaged over the VCH box is extracted and then correlated with winter SST anomalies for each 1° × 1° grid in the North Atlantic. Correlations are calculated for zero to nine-year lags. Areas that exceed subjectively chosen threshold correlations that decrease as the lag increases so as to maximize a propagation pattern are outlined for each lag in Fig. 4a. The resulting contours reveal a propagation pattern similar to that in Fig. 2 of Sutton and Allen^5^. However, if the 5-year running mean filter is removed before calculating the lagged correlations (Fig. 4b), the propagation pattern disappears. This implies that the apparent pathway in Fig. 4a results solely from the use of temporal smoothing. When we instead outline objectively chosen, statistically significant correlation thresholds (*P*<0.05) and do not use a running mean filter (Fig. 4c), years 0 and 1 encompass most of the western and central STG (STMW formation regions). STMW temperature anomalies have been hypothesized to resurface in the following winter due to summer stratification capping the anomalies and protecting them from air–sea exchanges^13^,^24^. Our results support this hypothesis with the caveat that our cutoff for significance at 0- and 1-year lag was relatively low (*r*≥0.36). Though the individual particles have Lagrangian integral timescales of <50 days, the STMW as a whole exhibits a 1-year SST memory due to particles entering and exiting the STMW during their lifetimes^12^,^13^. We also note that the STMW re-emergence region (Fig. 4c, green) is south of the GS/NAC indicated by either the GS north wall (Fig. 2) or the zero SSH contour (Fig. 4), both of which can be used to track the northern extent of the STG^25^. Thus, not only are the thermal anomalies short-lived, they are also primarily constrained to the STG. We conclude that SST anomalies are not advected or propagated along the GS/NAC from the STG to the SPG.

To determine why an inter-gyre pathway for SST anomalies appears with the 5-year running mean filter, we correlate the monthly time series of the NAO, an index of the atmospheric pressure gradient between Iceland and the Azores, with the AVHRR satellite SST (Fig. 4a). The spatial expression of the NAO forms a tri-pole pattern across the North Atlantic, with the SPG in phase with the tropical Atlantic and out of phase with the STG. This spatial pattern, combined with NAO timescales of roughly 2–7 years, gives context to the lagged correlation contours in Fig. 4a. With the 5-year running mean filter, the correlation contours of lags 0 through 5 years occur in the STG, where NAO-induced SST variability is in phase with the SST of the VCH box. For lags of 6 through 9 years, the correlations shift into the SPG where the NAO forcing is out of phase with the VCH SST. This shift into the SPG results not from the advection of water from the STG, but from the changing phase of the NAO and its opposing effect on the two gyres^26^. In addition, the 5-year running mean filter artificially increases the magnitude and prolongs the duration of the correlations that appear without the smoothing; the original 1-year memory of STMW SST in Fig. 4c is extended to 5 years by the temporal smoothing in Fig. 4a. Taken together, these results imply that the appearance of a SST anomaly pathway from the GS to the SPG in Fig. 4a is an artefact of the 5-year running mean filter introducing memory into NAO-induced SST oscillations rather than a signal of SST anomalies propagating along the GS/NAC^16^,^27^. Though other studies have pointed to the divergences and convergences in oceanic heat transport due to NAO forcing as a means to generate the SST anomalies in the GS region^16^, we propose that the apparent propagation pathway results from a smoothing window (5 years) that is on the same timescale as the NAO forcing (2–7 years).

Though for decades it has been assumed that waters from the surface GS flow northward as part of the upper limb of the AMOC, building on a recent study^11^ we demonstrate here that this throughput is not achieved by a surface connection. Importantly, we show that surface thermal anomalies generated in the subtropics are not transmitted to the surface waters at higher latitudes on interannual timescales. This work has implications for our understanding of how the AMOC achieves its poleward transport of heat.

## Additional information

**How to cite this article**: Foukal, N. P. & Lozier M. S. No inter-gyre pathway for sea-surface temperature anomalies in the North Atlantic. *Nat. Commun.* 7:11333 doi: 10.1038/ncomms11333 (2016).
